# Network analysis for science and technology management: Evidence from tuberculosis research in Fiocruz, Brazil

**DOI:** 10.1371/journal.pone.0181870

**Published:** 2017-08-08

**Authors:** Bruna de Paula Fonseca e Fonseca, Marcus Vinicius Pereira da Silva, Kizi Mendonça de Araújo, Ricardo Barros Sampaio, Milton Ozório Moraes

**Affiliations:** 1 Centro de Desenvolvimento Tecnológico em Saúde (CDTS), Fiocruz, Rio de Janeiro, Brazil; 2 Casa de Oswaldo Cruz (COC), Fiocruz, Rio de Janeiro, Brazil; 3 Instituto de Informação Científica e Tecnológica em Saúde (ICICT), Fiocruz, Rio de Janeiro, Brazil; 4 Diretoria Regional de Brasília (DIREB), Fiocruz, Brasília, Brazil; 5 Vice-Presidência de Ensino, Informação e Comunicação, Fiocruz, Rio de Janeiro, Brazil; 6 Laboratório de Hanseníase, Instituto Oswaldo Cruz (IOC), Fiocruz, Rio de Janeiro, Brazil; Institut Català de Paleoecologia Humana i Evolució Social (IPHES), SPAIN

## Abstract

Collaborative networks are of great value for science and technology (S&T) institutions as a way of sharing, generating and disseminating new knowledge that could ultimately lead to innovations. Driven by the need to assess the contribution and effectiveness of these networks in informing S&T management, we explored the evolution and dynamics of tuberculosis scientific networks involving the Oswaldo Cruz Foundation (Fiocruz), the major public health S&T Institution in Brazil. Social network analysis (SNA) was used to produce a 10-year (2005–2009, 2010–2014) retrospective longitudinal mapping of Brazilian tuberculosis research networks within the country and internationally, highlighting Fiocruz collaborations. Co-authorship analysis showed a significant expansion of collaboration in Brazil and the role of Fiocruz and other leading national institutions in maintaining connectivity, facilitating knowledge exchange and reducing network vulnerability. It also identified influential researchers that can act as information leaders and support strategic decisions. When we focused on networks inside the institution, the analysis showed a clear discontinuation between the clinical and the public health research areas, which needs specific internal policies to improve collaborations since outcomes in TB are expected to provide better diagnostic tools and more effective treatments. The approach provides evidence to support S&T management by pinpointing: key central institutions maintaining network connectivity; most influential researchers that can act as advisors/experts for investment and induction policies; key Fiocruz researchers that could improve information exchange, systems integration and innovation within the institution; opportunities for synergy between internal research groups working in complementary areas. In summary, we observed that SNA parameters proved to be a valuable tool that, along with other indicators, can strengthen knowledge platforms to support S&T management efforts.

## Introduction

Health innovation networks are considered essential for strengthening research capacities in developing countries, where human resources and funding are limited and research infrastructure is fragmented [1,2]. The Brazilian government has encouraged the establishment of research networks to address strategic health issues in response to increasing health and social demands [3]. This has prompted the development of indicators to evaluate the dynamics and effectiveness of collaborative networks in accessing knowledge and information towards innovation that could bring important insights for policy-makers in research management and strategic planning processes.

The analysis of scientific and technological (S&T) networks can provide useful information for monitoring and evaluating research productivity, decision-making processes and assessing institutional development [4]. The study of co-authorship networks through social network analysis (SNA) is a valuable tool for analyzing S&T collaborations and partnerships, providing input for research policy [5]. It has been used to study the dynamics of interdisciplinary sciences [6], evaluate the growth and structure of collaboration within academic communities [7], study the impact of collaboration on the performance of public research institutions [8] and support the performance assessment and development of health S&T organizations [9]. It enables the visualization of connections among actors in both, external and internal environments, providing a way to address intangible phenomena, such as knowledge flows, information sharing and communication [10]. Mapping the pattern of information flow across functional groups can yield critical insight to promote collaboration that will provide strategic benefit.

SNA has also been applied to understand collaboration networks in neglected tropical diseases, generating evidence to guide policy-planning efforts in Brazil, Canada and Germany [11–15]. Noteworthy, Vasconcelos & Morel (2012) showed that the tuberculosis (TB) research community in Brazil is split in two polar groups: basic research and product development, and emphasized the importance of carrying out these analyses in countries like Brazil, which have recently consolidated its scientific base [12]. In fact, attempts to bridge basic science and patent applications along with a close dialogue with the Brazilian Ministry of Health have resulted in the establishment of a Brazilian TB research network (REDE-TB) [16]. In 2014, the World Health Assembly recognized Brazil as one of the few countries where the presence of a TB research network diminished this gap, improving integration of academia, policy makers and industry, fostering human resources training and product development committed to the TB research field [17]. Nevertheless, a permanent evaluation of the impact of these strategies needs to be pursued.

Brazil is one of the 22 high burden TB countries in the world [18]. The emergence of HIV-infected individuals and multidrug resistant *Mycobacterium tuberculosis* strains maintain the disease as a great public health threat, with high levels of mortality and morbidity on BRICS countries [17]. The World Health Organization has developed a Global Action Framework for TB Research to foster high-quality research, encouraging the development of regional and international networks for research and capacity building with a special emphasis on low- and middle-income countries carrying the largest disease burden [19]. TB is recognized as a high burden public health issue for which research is critically needed to develop early and accurate diagnosis and efficient treatment.

The Oswaldo Cruz Foundation (Fiocruz) is considered one of the most important public health institutions in the world, being engaged in a range of activities including, product research and development, technological innovation, healthcare, production, education and training, information and communication, quality control and implementation of social programs. Fiocruz has institutes in 10 Brazilian states and an international office in Maputo, Mozambique. It has 12,000 people workforce in permanent positions and supports students in graduate programs. Its TB portfolio of activities includes efforts across a continuum from fundamental research to clinical, epidemiological, implementation, health system, product development, social science research, and services for prevention, diagnosis and treatment. As Fiocruz covers all the TB production chain, being an integrant part of the Ministry of Health, it was considered an appropriate model to apply SNA as a tool to guide science and technology (S&T) management.

In this paper, SNA was applied to generate evidence on the evolution of scientific connectivity in TB research, highlighting the role of Fiocruz in this process, its external and internal relations. Through SNA, central institutions in TB research that had a role in maintaining connectivity, facilitating knowledge exchange and reducing network vulnerability could be identified. Influential researchers that could act as advisors/experts for investment and induction policies were also recognized. At Fiocruz, key researchers that could improve information exchange, systems integration and innovation within the institution were identified, as well as opportunities for synergy between research groups working in complementary areas. The paper aims to contribute to the discussion on how SNA can support S&T management.

## Data and methodological approach

### Data collection and search strategy

TB publications by Brazilian-based scientists (as authors or co-authors) were retrieved from the Web of Science Core Collection (WoS) database for the period of 2005 to 2014 (n = 1,934). The query was directed to the title, abstract and keywords using the search terms “tuberculosis, tuberculoses or “Koch* disease” or “Koch's disease” or antitubercular or tuberculostatic”, and filtered by country (Brazil). Only published or in press articles were included in the analysis. In addition, for comparative purposes, data were collected on global scientific publications on TB (n = 41,171).

### Cleaning and standardization of data

The data was imported from the WoS into the data/text mining software VantagePoint (Search Technology Inc). Duplicate records and scientific papers addressing species of mycobacteria other than *Mycobacterium tuberculosis* or other related pathogens were excluded from the dataset (n = 241).

Three publications with 100 or more authors were excluded from the analysis. In most cases, a large number of co-authors is a consolidation of independent contributions of multicentric studies, rather than a joint collaborative intellectual effort [20]. The final sample consisted of 1,690 papers (S1 File).

Names of institutions and authors were standardized to ensure the correct acknowledgement of their scientific production. The data was processed with the “list clean up” function of the VantagePoint software on the “author affiliations” and “author full name” fields. For researchers with more than one professional affiliation, it was assumed that these individuals act as collaboration link between these institutions [21].

### Bibliometric mapping and clustering

A combined approach of mapping and clustering was used to provide an overview of the research themes in the set of retrieved publications. Bibliometric maps were constructed using the VOS (visualization of similarities) mapping technique available on the VOSviewer software [22]. Based on the number of co-occurrences of terms in the title or abstract of the same publication, the software estimates their “similarity” (affinity) using the “association strength” measure proposed by Van Eck and Waltman [23]. The larger the number of publications in which two terms co-occur, the stronger the terms are considered to be related to each other. Therefore, terms that often co-occur in the same publications are located close to each other in a “term map” and less strongly related terms (low co-occurrence) are located further away from each other. Graphically, each term is represented by a circle, where its diameter and label size indicate the number of occurrences of the corresponding term in the title or abstract of publications. To identify clusters of related terms, the software uses a weighted and parameterized variant of modularity-based clustering [24].

### Social network analysis: Network assembly, metrics and visualization

SNA is a theoretical approach that uses a set of techniques to understand and quantify the relationship between members of a network (nodes or actors), which can be individuals, groups, institutions and even whole countries [25]. SNA includes indicators/metrics that may reflect the properties of the network as a whole or of its individual nodes. The network-level indicators provide information on its overall structure and properties, such as size and connectivity. Indicators at the individual level describe the importance of a particular node relative to all other nodes, based on the nature of its interactions [26].

The cleaned data was formatted into adjacency matrixes by VantagePoint to map co-authorship relationships between institutions and researchers. Matrixes were imported into the open-source software Gephi [27] for network visualization. As co-authorship requires reciprocal cooperation among the participants, all connections have been considered as non-directional.

The TB research networks involving Fiocruz researchers were analyzed in three levels: i) interinstitutional collaboration among national and international organizations; ii) individual collaboration with national and international partners; and iii) intrainstitutional collaboration, represented by Fiocruz's internal network of researchers.

The analysis of the evolution of connectivity/cohesion of the interinstitutional network was carried out in two five-year periods (2004–2009 and 2010–2014). During this period of collaboration, it is assumed that the exchange of information happens more intensely [28].

Statistical analysis was done with Gephi and UCINET software [29]. The following metrics were used to characterize the institutional networks structure [30]: i) number of nodes; ii) number of links; iii) size of the giant component; iv) average degree; v) average clustering coefficient; vi) average path length; vii) connectivity and fragmentation; and viii) E-I index (S1 Table).

The identification of influential strategic institutions and researchers in the network was based on the calculation of following key centrality measures [31]: i) degree centrality; and ii) betweeness centrality. Central institutions generally have more access and control over resources, leading the knowledge exchange and preventing many groups from isolation [32,33]. Consequently, these institutions are often associated with innovative activities [34].

The above-mentioned indexes were calculated and normalized according to the network size. The definitions and meaning of these indicators in the context of this study are presented in S1 Table.

## Results

### Fiocruz accounted for nearly a quarter of Brazilian research on tuberculosis

**Fig 1:**
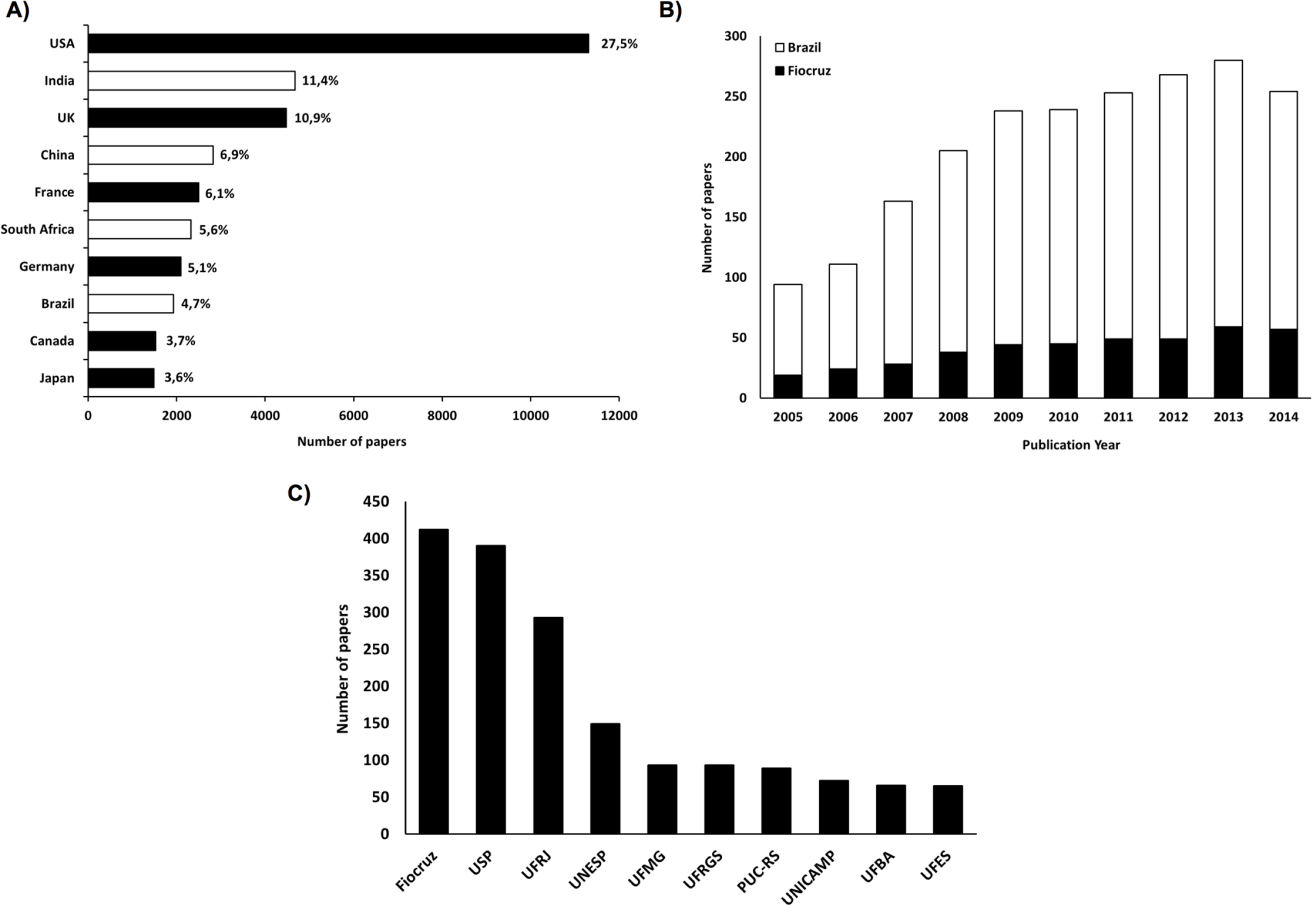
Tuberculosis research publications (2005–2014). **A)** Top ten countries with highest number of articles published and relative contributions (%). White bars indicate high TB burden countries. **B)** Number of papers published by Brazilian authors by year. **C)** Top ten Brazilian institutions with highest number of articles. Fiocruz: Oswaldo Cruz Foundation; USP: University of São Paulo; UFRJ: Federal University of Rio de Janeiro; UNESP: São Paulo State University; UFMG: Federal University of Minas Gerais; UFRGS: Federal University of Rio Grande do Sul; PUC-RS: Pontifical Catholic University of Rio Grande do Sul; UNICAMP: State University of Campinas; UFBA: Federal University of Bahia; UFES: Federal University of Espírito Santo.

Brazil accounted for approximately 5% of the world’s scientific publications on TB, ranking at the 8th position globally. When compared to other countries with high disease burden, such as India, China and South Africa, Brazil ranks the 4^th^ place among these countries in number of papers published (Fig 1A).

Fiocruz and Brazil overall had a similar publication trend, with a growth from 2000–2009 followed by a relatively stable production (Fig 1B). Among a total of 1,112 institutions identified, Fiocruz accounted for approximately 24% (n = 412) of TB publications involving Brazilian institutions, followed by the University of São Paulo (n = 388) and the Federal University of Rio de Janeiro (n = 293) (Fig 1C).

Although Fiocruz has scientific institutes in ten states of the country, the headquarters in Rio de Janeiro accounted for 79% of all TB publications in the organization (n = 326). Other Fiocruz institutes involved in TB research were located in the states of Pernambuco (8.2% of all Fiocruz publications), Bahia (5.3%), Minas Gerais (4.1%), Amazonas (2.9%), Mato Grosso and Paraná (0.2% each one). The institutes of Rio de Janeiro and Pernambuco had the largest number of joint publication (5 papers).

### Fiocruz research is aligned with the main fields of tuberculosis research in Brazil

**Fig 2:**
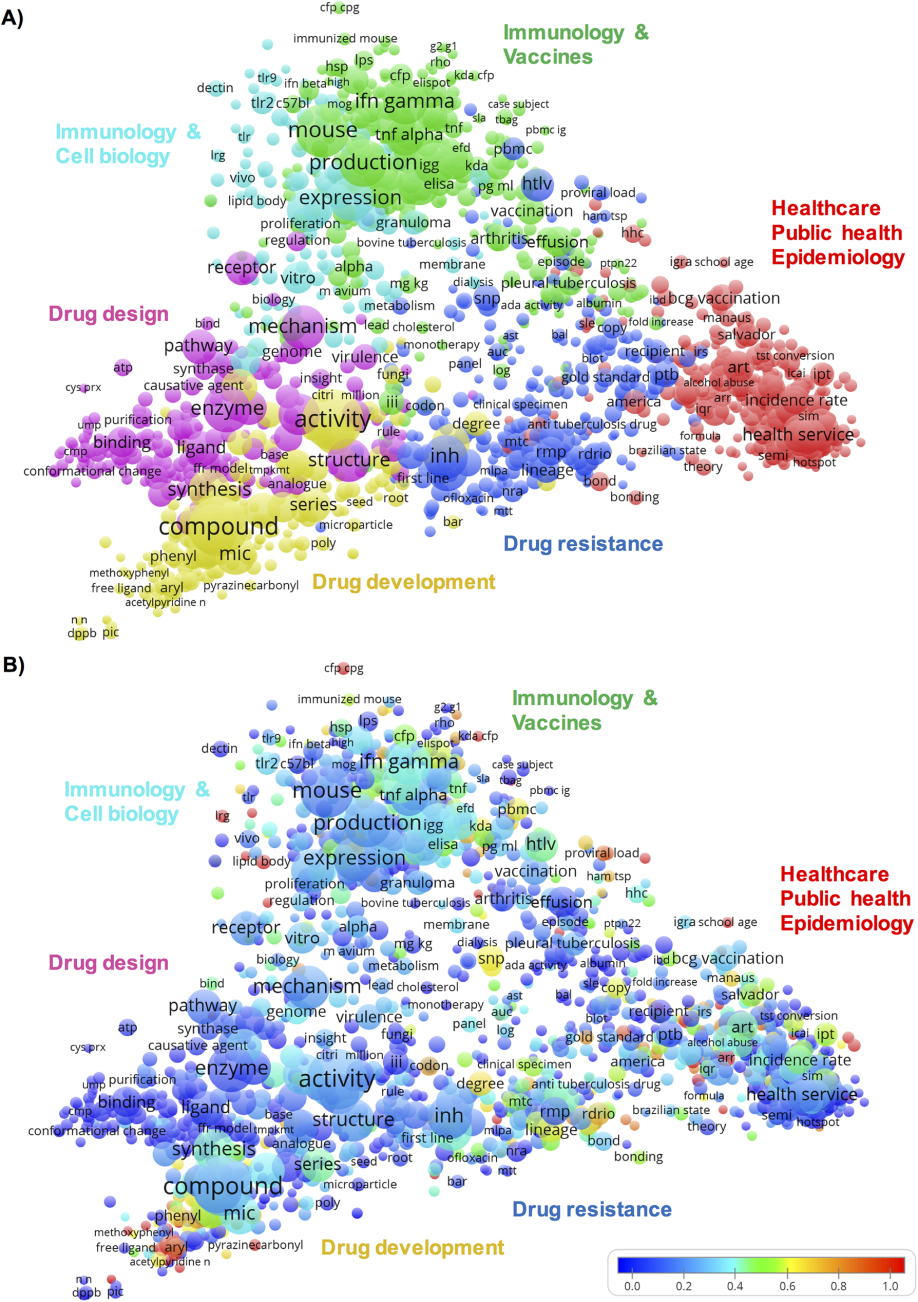
Term map of Brazilian TB research. The map shows 1,651 terms extracted from titles and abstracts of TB publications involving Brazilian institutions. The closer two terms are located to each other, the stronger their relation. Each term is represented by a circle and its diameter and label size indicate the number of publications that have the corresponding term in their title or abstract. Each term occurs in at least five publications. **A)** Colors indicate clusters of terms that have co-occurred more frequently in the dataset. **B)** Colors indicate the occurrence of a term in publications involving Fiocruz relative to Brazil’s average, where blue represents a low occurrence, green average, and red a high occurrence.

The map of all TB publications involving Brazilian organizations is shown in Fig 2A. The terms displayed on the map were grouped in six clusters illustrated in different colors. Each cluster represents a research area, identified by its most frequent terms related to TB research. Starting from the bottom (dark blue cluster) and moving clockwise these were: i) Drug resistance (mutation, resistant strain, isonicotinylhydrazide—inh); ii) Drug development (compound, activity, synthesis, minimal inhibitory concentrations–mic); iii) Drug design (enzyme, inhibitor, interaction, structure); iv) Immunology & Cell biology (macrophage, expression, bacterium, activation); v) Immunization & vaccines (mouse, IFN-gamma, antigen, BCG, vaccine); vi) Healthcare, Public health & Epidemiology (health service, municipality, incidence rate).

Publications from Fiocruz researchers are depicted in Fig 2B, representing an overlay visualization of Fig 2A. In this research map, blue represents a lower score, green an average, and red a higher score of occurrence of a term in Fiocruz’s publications in relation to Brazil’s average. Areas in which Fiocruz’s researchers have published in the past 10 years included: i) Drug resistance; ii) Drug development; iv) Immunology & Cell biology v) Immunology & Vaccines; and vi) Healthcare, Public health & Epidemiology. Drug design was an area with very limited contribution of Fiocruz.

### Fiocruz is a strong player in the Brazilian TB research network

**Fig 3:**
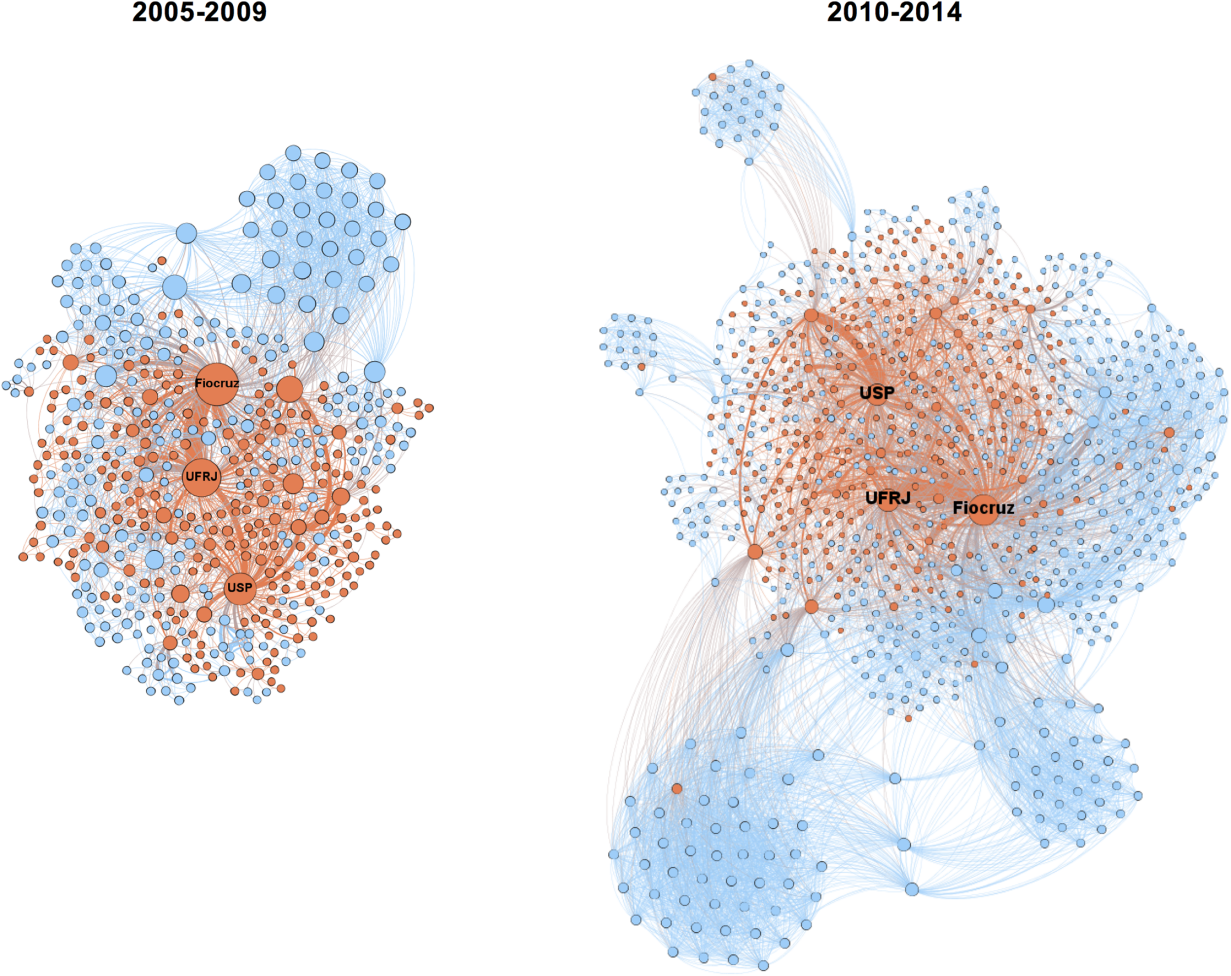
Evolution of TB research networks involving Brazilian institutions (2005–2009 and 2010–2014). Each node represents one institution and two institutions were considered connected if their members shared the authorship of a paper. Nodes are color coded—red for Brazil and blue for foreign organizations. The size of the nodes is proportionate to their degree centrality. For visualization purposes only the giant component is shown. The top three Brazilian organizations with highest degree centrality are labeled.

The evolution of the Brazilian TB research network was mapped based on articles retrieved in two quinquennials: 2005 to 2009 (S2 File) and 2010 to 2014 (S3 File) (Fig 3).

During the first five-year period, the network included 239 (49.5%) national institutions and 243 (50.4%) international institutions. The following period involved 336 (37.9%) Brazilian institutions and 549 (62.2%) foreign institutions. A total of 87 countries were involved during the full period. United States, England and France-based institutions were the most frequent international partners of Fiocruz, sharing authorship in 18%, 13% and 7% of all articles, respectively. Fourteen countries with high disease burden collaborated with Fiocruz, but their association was less frequent. Among these countries, South Africa was the partner with the highest number of articles in co-authorship with Fiocruz (13 articles or 3%).

The evolution of the structure of the Brazilian TB research network was evidenced through the cohesion/connectivity indicators presented in Table 1.

**Table 1:** Connectivity indicators of the TB research network involving Brazilian institutions (2005–2014).

Indicator	Period	
	2005–2009	2010–2014
Number of nodes (institutions)	482	885
Number of links	2,486	7,154
Number of components	15	16
Giant component size	96.0%	97.6%
Average degree*	10.7	16.5
Connectivity	92.3%	95.3%
Fragmentation	7.7%	4.7%
Average clustering coefficient*	0.828	0.851
Average path length*	2.809	2.739
E-I index (Brazilian institutions)*	-0.349	-0.221
E-I index (Fiocruz)*	0.187	0.260

*Calculated on the giant component

The Brazilian TB research network has grown, almost doubling its size, from the first to the second 5-year-period, over the 10 years evaluated. This fact, together with the increase in the average degree, size of the giant component, average clustering coefficient and connectivity, indicated strengthening of network cohesion over the years. These characteristics associated to the decrease in average path length indicate that the structure of this network was potentially very efficient in generating knowledge (high connectivity) and sharing and diffusion of knowledge (low distance).

The negative E-I index indicated an increasing cooperation with national organizations. The increase of Fiocruz E-I index is an indication of a slight increase in international collaboration from the first to the second 5 years.

Centrality analysis allowed the identification of the most influential institutions in each period (Table 2).

**Table 2:** Top three most influential Brazilian institutions in the national TB research network according to centrality measurements.*

	2005 to 2009			2010 to 2014		
	Rank	Institution	Value	Rank	Institution	Value
**Degree centrality**	1	Fiocruz	0.335	1	Fiocruz	0.369
	2	UFRJ	0.292	2	UFRJ	0.249
	3	USP	0.235	3	USP	0.228
**Betweeness centrality**	1	Fiocruz	0.239	1	Fiocruz	0.299
	2	USP	0.235	2	USP	0.206
	3	UFRJ	0.210	3	UFRJ	0.164

Fiocruz: Oswaldo Cruz Foundation; USP: University of São Paulo; UFRJ: Federal University of Rio de Janeiro

*Analysis considered only the institutions included in the giant component

Fiocruz had a prominent role in TB research in Brazil in the two periods evaluated. High degree centrality indicates a high number of direct connections or collaborations and high betweeness centrality suggests that the institution functioned as a bridge between groups that in its absence would be disconnected. This advocates Fiocruz as an influential player in TB research, which can facilitate access to new information and resources, enabling knowledge transfer.

The University of São Paulo (USP) and the Federal University of Rio de Janeiro (UFRJ), were also key players in the network. As central organizations, they probably had a role in maintaining network cohesion/connectivity, ensuring that less connected or peripheral organizations had access to new knowledge and information.

The most frequent partners of Fiocruz were national universities, including the Federal University of Rio de Janeiro (UFRJ) and the Federal University of Pernambuco (UFPE), with 131 (32%) and 32 (7%) articles in collaboration. Most frequent international collaborators were the Johns Hopkins University (USA) and the University of London (England).

### Fiocruz researchers are among the most influential in the Brazilian TB research network

Influential researchers from Fiocruz were identified in the co-authorship network built with all articles retrieved from 2005 to 2014 (S4 File). Their individual degree and betweeness centralities are shown in Table 3. The Brazilian TB research network involved 6,400 researchers, among first authors and coauthors, including 439 researchers from Fiocruz (6.9%). Twenty-three percent of these researchers published one or more papers on TB per year. The affiliation of these researchers was confirmed by cross-checking institutional records. For this particular analysis, visiting researchers and research students were excluded, resulting in 208 researchers formally employed in the institution.

**Table 3:** Top ten most influential scientists in the national TB research network according to the degree and betweeness centrality indexes.*

Indicator	Rank	Researcher	Affiliation	Value	Number of publications
**Degree centrality**	1	Researcher A	UFRJ	0.074	72
	2	Researcher B	UNESP	0.052	70
	3	**Researcher C**	**Fiocruz**	0.050	32
	4	**Researcher D**	**Fiocruz**	0.042	63
	5	Researcher E	UNESP	0.040	50
	6	Researcher F	FEPPS	0.039	34
	7	Researcher G	USP	0.038	49
	8	**Researcher H**	**Fiocruz**	0.036	18
	9	Researcher I	USP	0.033	56
	10	Researcher J	PUC-RS	0.029	66
**Betweeness centrality**	1	Researcher A	UFRJ	0.201	72
	2	**Researcher D**	**Fiocruz**	0.097	63
	3	Researcher B	UNESP	0.096	70
	4	Researcher G	USP	0.080	49
	5	Researcher I	USP	0.076	56
	6	Researcher L	UFMG	0.056	14
	7	Researcher M	UFRJ	0.054	20
	8	**Researcher C**	**Fiocruz**	0.051	32
	9	Researcher N	IAL	0.045	16
	10	Researcher O	UNIFESP	0.039	9

Fiocruz: Oswaldo Cruz Foundation; USP: University of São Paulo; UFRJ: Federal University of Rio de Janeiro; UNESP: São Paulo State University; FEPPS: State Foundation for the Production and Health Research; PUC-RS: Pontifical Catholic University of Rio Grande do Sul; UFMG: Federal University of Minas Gerais; IAL: Adolfo Lutz Institute; UNIFESP: Federal University of São Paulo.

*Analysis considered only the researchers included in the giant component

Three TB researchers from the Oswaldo Cruz Institute (IOC) of Fiocruz, Rio de Janeiro, were among the ten most influential scientists in the Brazilian network. They have a high number of connections with other researchers and work in the areas of applied microbiology and genetics of microorganisms, also acting as intermediaries of the information flow.

Although degree centrality expresses collaboration in the network, it does not necessarily reflect the number of publications. Researcher C, for example, has a high degree centrality in the network, but has less publications than Researcher D (Table 3). This means that Researcher C has a stronger outreach in collaboration, as most of his/her articles were published in co-authorship with other researchers.

### Internal network analysis revealed opportunities for synergy between research groups

**Fig 4:**
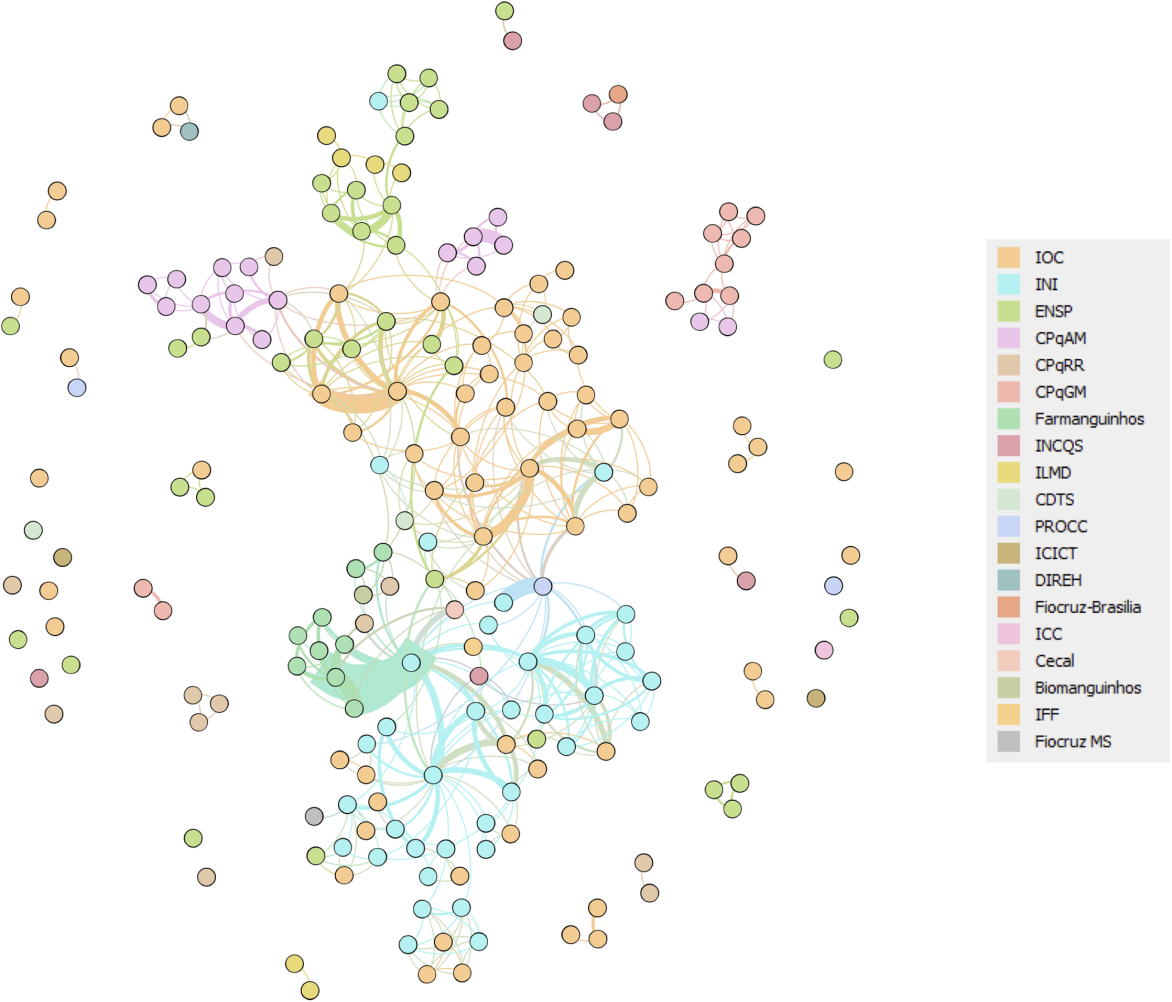
Intrainstitutional TB research network in Fiocruz (2005–2014). Each node is a researcher and two researchers were considered connected if they shared the authorship of an article. Researchers were color coded according to the technical/scientific or administrative unit to which they belong in Fiocruz. The thickness of the lines represents frequency of collaborations between the researchers. IOC: Oswaldo Cruz Institute; INI: National Institute of Infectology Evandro Chagas; ENSP: National School of Public Health Sérgio Arouca; CPqAM: Aggeu Magalhães Research Center (Fiocruz Pernambuco); CPqRR: René Rachou Research Center (Fiocruz Minas Gerais); CPqGM: Gonçalo Muniz Research Center (Fiocruz Bahia); Farmanguinhos: Institute of Drugs Technology; ILMD: Leônidas and Maria Deane Institute (Fiocruz Amazônia); INCQS: National Institute for Quality Control in Health; CDTS: Center for Technological Development in Health; PROCC: Scientific Computing Program; ICICT: Institute of Communication and Scientific Information and Technology in Health; ICC: Carlos Chagas Institute (Fiocruz Paraná); IFF: Fernandes Figueira Institute; Fiocruz MS: Fiocruz Mato Grosso do Sul; Biomanguinhos: Immunobiological Technology Institute; Cecal: Laboratory Animal Breeding Center; DIREH: Directorate of Human Resources.

The assessment of the internal Fiocruz co-authorship network took into account all records retrieved from 2005 to 2014, looking at the extent to which researchers were embedded into the institutional structure and opportunities for improvement of their integration (Fig 4). In this network, only the 208 researchers formally employed by Fiocruz and their co-authorship relations were represented. Each node is a Fiocruz researcher and two researchers were considered connected if they shared the authorship of a paper. Researchers were color coded according to the technical/scientific or administrative unit to which they belong in Fiocruz.

The IOC was the unit with the largest number of researchers in the network (31.4%), followed by the National Institute of Infectious Diseases Evandro Chagas—INI (16.9%) and the National School of Public Health Sérgio Arouca—ENSP (16.4%). At Fiocruz, the IOC acts in the area of research, technological development and innovation, also providing diagnostics services for infectious and genetic diseases. ENSP is focused on training and development of human resources for the Unified Health System (SUS), being specialized in the analysis and discussion of health problems of the Brazilian population. The INI is focused on clinical research and provision of healthcare services in infectious diseases.

Some researchers were not connected to the giant component, indicating they have no joint publications with other members of the network. This is a sign of a possible fragmentation of Fiocruz's TB research and an opportunity for promoting integration. More collaboration was seen between researchers working in the same technical/scientific Institute. This is especially true for researchers based in different geographical regions in Brazil, such as CPqAM (Manaus) and CPqGM (Bahia).

**Fig 5:**
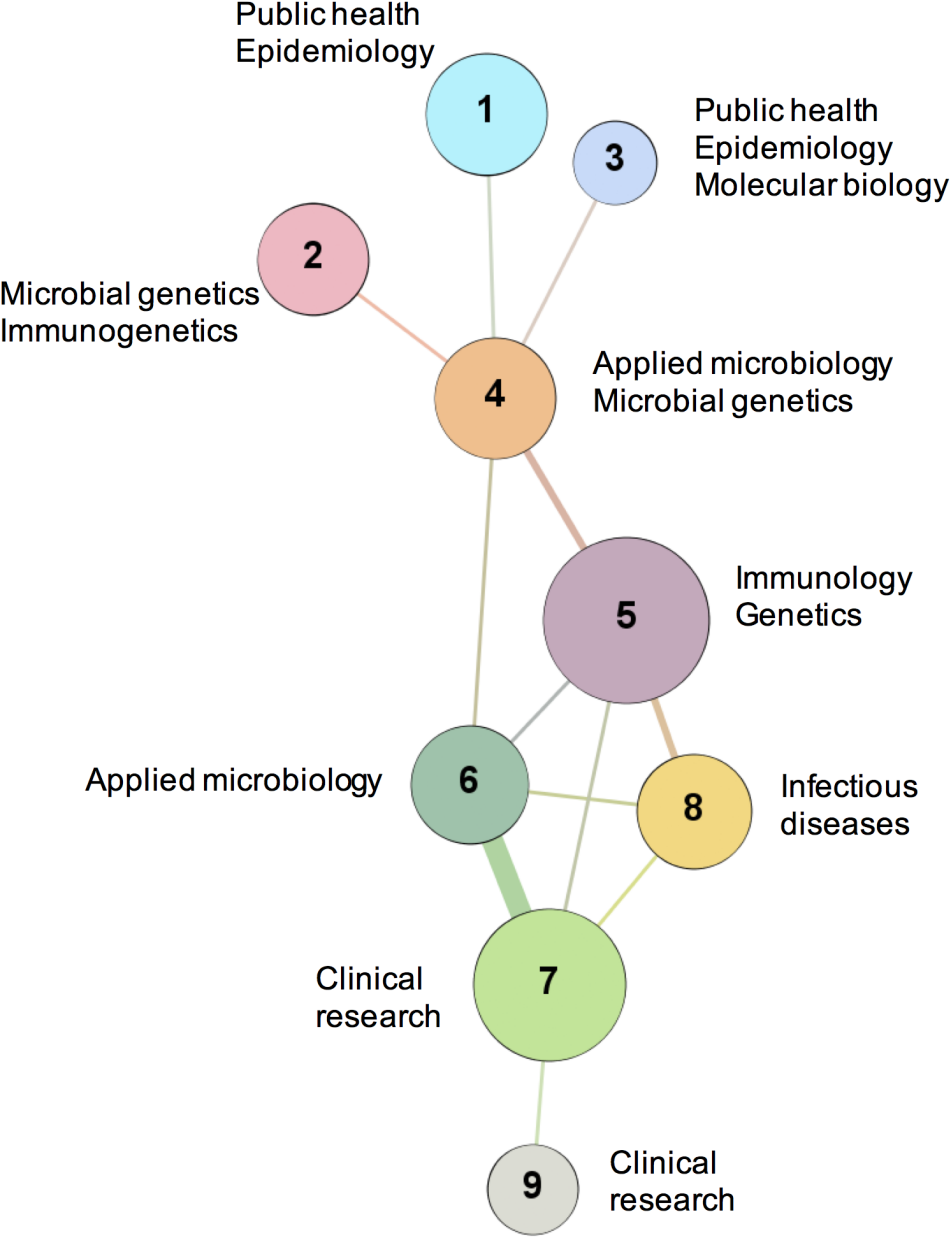
TB network and research communities within Fiocruz (2005–2014). Each node is a community formed by researchers affiliated with Fiocruz. Two communities were considered connected if their members shared the authorship of an article. The size of the nodes is proportional to the number of members of the community. The thickness of the lines indicates the frequency of collaboration. For visualization purposes only the giant component is shown. The communities were numbered from 1 to 9.

The community structure of Fiocruz’s network was reviewed using the algorithm proposed by Blondel et al. [35]. The algorithm identifies a community when the number of connections in a particular group of nodes is greater than the number of connections expected between them and the rest of the network. Belonging to a community indicates that the researchers within it collaborate more within the community than with researchers outside the group. There were nine different communities in the Fiocruz giant component. For each community, the main research areas were identified according to the areas of activity of their researchers, as included in the Lattes curriculum vitae online platform of the Brazilian Council for Scientific and Technological Development (CNPq) (Fig 5).

The community structure of the Fiocruz intrainstitutional network showed a distance between communities 7, 8 and 9, whose main area is clinical research, and communities 1 and 3, whose research area is public health. It also shows the integrating role of communities 4, 5 and 6, which are characterized by applied microbiology, immunology and genetics, and are functioning as "bridges" between these otherwise disconnected areas.

## Discussion

In this study, the evaluation of co-authorship networks identified structural patterns of TB research involving Brazilian scientists. Brazil accounted for approximately 5% of the world’s scientific publications on TB and Fiocruz was responsible for nearly a quarter of Brazilian research. Research for TB elimination requires an intensification of efforts across a continuum from fundamental research to clinical, epidemiological, implementation, health system, and social science research [36]. Research areas addressed by Brazilian and Fiocruz’s researchers are aligned with this recommendation, probably as effect of multidisciplinary projects fostered by the government [3].

Biomedical research is crucial to the development of new tools and strategies for prevention, diagnosis and cure, and collaboration between researchers is a key component to accomplishing this goal [37]. Our findings suggest that Brazilian research institutions are embedded in highly connected networks, strengthened through the years. This increased scientific cooperation is in line with the worldwide trend in all areas of science [20], particularly in medical, biological and biomedical research [38]. This is also consistent with efforts envisaged towards training and integration of TB researchers by the Brazilian government.

Although the involvement of international institutions in the network increased in the second period evaluated, the decrease in the E-I index indicated that national institutions cooperate more frequently between each other than with foreign institutions. In fact, researchers are naturally more likely to collaborate when working in the same geographic region, especially as the exchange of knowledge is facilitated by physical proximity [39,40]. This also suggests that training and capacity building created by networks such as REDE-TB were key towards the strengthening of TB collaborative research. Nevertheless, collaboration with high burden countries has to be wired, as it could improve access to local knowledge and better understanding of the disease in different endemic contexts.

Centrality analysis of the interinstitutional network highlighted the role of Fiocruz in the dissemination of knowledge in TB, through collaborations. High degree centrality from Fiocruz indicated a strong collaborative pattern in research. Together with UFRJ and USP, Fiocruz probably had a vital role in maintaining the connection between the overall research network and in ensuring that less well connected organizations gained access to new knowledge and information on TB, a feature that contributed to reduce the network vulnerability.

Centrality analysis of the individual network identified the most influential Brazilian researchers. Most of them had at least 10 years of TB work, which certainly would give them a "preferred connection". New researchers would preferentially seek collaboration with well-established and connected researchers in the network [41]. In fact, it has already been shown that high degree and betweeness centralities play an important role in the preferential co-authorship connection of new actors [42]. As these researchers have great access to resources and new information, they can act as advisors/experts in investment and induction policies for the Brazilian government.

Among the 6,400 individuals, three Fiocruz researchers had an influential role in the Brazilian network for TB research. For Fiocruz, these researchers can be sources of information on technological trends and identify potential partners for research, making the network more connected and productive. A core group of highly connected researchers can improve information exchange, systems integration and innovation within Fiocruz. Their experience can guide strategic investment in new technologies and in product development. Additionally, researchers with high betweeness centrality can also serve as "agents of change" [43], being able to identify projects that can be executed in partnership and integrate disconnected members into the network.

The identification of non-connected researchers working in common areas within Fiocruz is a point of concern. Their integration is a way of avoiding duplication of scarce resources and means. Community structure shows how much a group relates to each other in the network. In Fiocruz, there is a clear separation between the ​​clinical research area and the public health area, reflecting limited interaction within TB research field. Collaboration among researchers is influenced by several factors, including personal compatibility, work connections (interests and skills), incentives (motivation), and socio-technical infrastructure [44]. Collaboration relies heavily on people making personal connections, but this may take time as well as infrastructure that promotes awareness of research capacities and facilitates broad, or unlimited, access to colleagues. As multidisciplinarity increases and accelerates the success of innovations [45], interaction between different research areas should be promoted for a more integrated and broad view of projects, adding different perspectives to TB research at Fiocruz. Institutional incentives through courses, workshops, meetings, development of joint projects for specific calls for proposals can be important means of fostering such cooperation.

We recognize the limitation of the use of co-authorship data as an indicator of scientific collaboration. Still, it is assumed that in most cases co-authorship indicates active cooperation, in addition to the simple exchange of material or information. Also, although WoS covers more than 12,000 scientific journals and has been widely used in the study of Brazilian research institutions [46,47], it is possible that some national, regional or specialized journals were not included in the database.

## Conclusions

Network analysis proved to be a useful mechanism for assessing collaboration performance and supporting S&T management in research institutions by pinpointing: (1) key central institutions maintaining network connectivity; (2) most influential researchers that can act as advisors/experts for investment and induction policies; (3) key Fiocruz researchers that could improve information exchange, systems integration and innovation within the institution; (4) opportunities for synergy between internal research groups working in complementary areas. Although S&T management cannot be based only on the collaborative pattern of an institution, as well as policies cannot be the only assessment to define strategies, the analysis of networks is a necessary reference to establish action plans and support institutional management decisions.

## Supporting information

S1 File Full dataset with all papers and related information analyzed in this study. (XLSX)

S2 File Organizational network matrix (2005–2009). (CSV)

S3 File Organizational network matrix (2010–2014). (CSV)

S4 File Researchers network matrix (2005–2014). (CSV)

S1 Table Definitions, metrics and meanings of the network indicators in this study. (DOCX)
