# Supplementary material for: Network analysis for science and technology management: Evidence from tuberculosis research in Fiocruz, Brazil
Source: PLoS One. 2017 Aug 8;12(8):e0181870. doi: 10.1371/journal.pone.0181870 (PMC5549698; doi:10.1371/journal.pone.0181870)
Supplement: S1 Table — (DOCX) [file pone.0181870.s005.docx]

**Table S1: Definitions, metrics and meanings of the network indicators in this study.**

| **Metrics** | **Definition** | **Meaning** |
| --- | --- | --- |
| **Network Size** | | |
| Number of nodes | Number of individual actors within a network. | Institutions in the co-authorship network. |
| Number of links | Measures the relationships or connections between actors. | Co-authorship between institutions. |
| **Network connectivity/cohesion** | | |
| Giant component | Largest subset of nodes in a network in which all of them are linked to each other, directly or indirectly. | Largest group of institutions connected through joint publications. The larger the giant component size, or percentage of institutions included within it, the more interconnected the network is. |
| Average degree | Average number of direct connections the network nodes have. | Average number of collaborations per institution. The higher the average degree, the more connected the network is. |
| Average clustering coefficient | Measures the extent to which the nodes in the network establish a perfect cluster, where all the nodes are connected between them. | The extent of full connectivity between institutions. A high average clustering coefficient indicates that more institutions are interconnected within the network. |
| Average path length | Average smallest number of connections that a node needs in order to reach any other in the network. | The average distance between institutions. The lower the average path length, the more direct is the connection between institutions. |
| Connectivity and fragmentation | The proportion of pairs of nodes that are connected (connectivity) or not connected (fragmentation) | The lower the value of fragmentation, the greater the connectivity and integration between institutions. |
| E-I index | Calculates the level of internal relations (homophilia) by measuring the number of connections with members of the same group (internal) vs connections with members of another (external) group. The index value varies from -1 (all connections are internal to the group) to 1 (all connections are external to the group). | The groups were defined according to their geographic location: Brazil or International. The lower the E-I index of the network, the more endogenous are the collaborations. The higher the E-I index of a Brazilian institution, the more internationalized is its pattern of cooperation. |
| **Centrality/significance of nodes in the network** | | |
| Degree centrality | Number of a node’s direct connections. | Degree centrality is a measure of the influence, access, or direct control that an institution/researcher has in relation to its contacts. A high degree centrality indicates more direct contact with many members of the network, making the institution or researcher a focal point of communication in the network. |
| Betweeness centrality | Indicates the extent to which a node acts as a “bridge” between the various other nodes in the network, who otherwise would be disconnected. | Measures how much an institution/researcher mediates the connection between other groups, functioning like "bridges". Institutions or researchers with high betweeness centrality are on the shortest path among other nodes very often. They are considered highly central because they control the flow of information in the network, connecting several groups. |
